# Complete resection of local advanced thymic carcinoma with total aortic arch replacement after chemotherapy: a case report

**DOI:** 10.1186/s40792-019-0713-7

**Published:** 2019-12-12

**Authors:** Hidenori Kuno, Soichiro Funaki, Kenji Kimura, Kazuo Shimamura, Keiwa Kin, Toru Kuratani, Yoshiki Sawa, Yasushi Shintani

**Affiliations:** 1 Department of General Thoracic Surgery, Osaka University Graduate School of Medicine, L5-2-2 Yamadaoka, Suita City, Osaka, 565-0871 Japan; 2 Department of Cardiovascular Surgery, Osaka University Graduate School of Medicine, Osaka, Japan

**Keywords:** Advanced thymic carcinoma, Multimodal therapy, Aortic arch replacement, Combined resection

## Abstract

**Background:**

Although complete surgical resection of thymic carcinoma is a prognostic factor, it is not always an option for advanced tumors because of locoregional invasion. Extended surgery combined with a major blood vessel procedure remains controversial because of the increased risk of mortality.

**Case presentation:**

Chest computed tomography (CT) uncovered an abnormal shadow in the mediastinum of a 74-year-old man. An irregularly shaped tumor obstructed the left innominate vein, and invasion of the aortic arch was suspected. A CT-guided percutaneous needle biopsy revealed squamous cell carcinoma of the thymus, which was considered unresectable. The patient underwent chemotherapy elsewhere, then was referred to us for surgical resection. We combined extended surgery with total aortic arch replacement under a cardiopulmonary bypass. Complete resection was achieved, and the patient remains alive without recurrence at 3 years after surgery

**Conclusion:**

Resection including aortic arch replacement might be an option that can achieve complete resection of local advanced thymic carcinoma.

## Background

Thymic carcinoma is a relatively rare tumor that accounts for 20% of all thymic epithelial neoplasms [1]. Unlike thymoma, thymic carcinoma tends to be aggressive, with the features of local invasion, intrathoracic lymphadenopathy, and distant metastases.

Complete surgical resection is generally a significant prognostic factor for survival. However, complete surgical resection is not always an option for treating advanced thymic carcinoma because of locoregional invasion [2]. Furthermore, extended surgery that includes major blood vessels remains controversial due to increased risk of mortality. Here, we describe complete resection of local advanced thymic carcinoma combined with total aortic arch replacement after chemotherapy.

## Case presentation

**Fig. 1:**
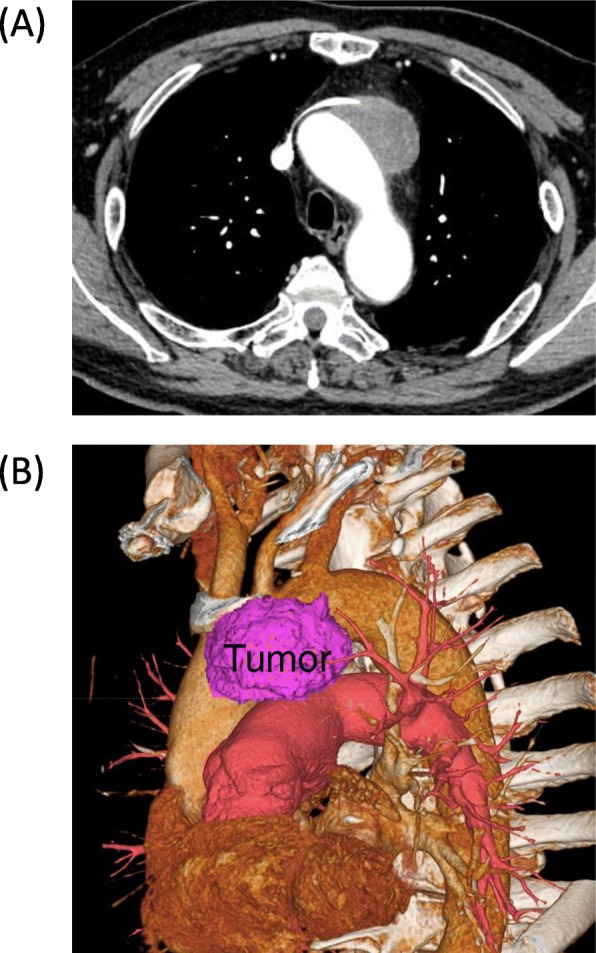
Preoperative radiographic images after chemotherapy. **a** Chest computed tomography image shows anterior mediastinal tumor, 55 × 55 × 50 mm, with AA invasion. **b** Three-dimensional image constructed using SYNAPSE VINCENT® (Fuji Film, Tokyo, Japan). AA, aortic arch; CT, computed tomography

**Fig. 2:**
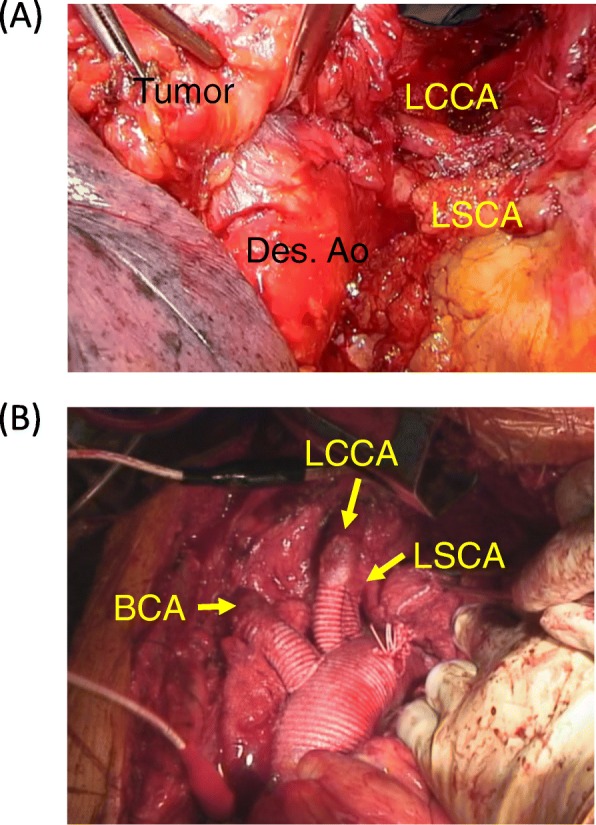
Intraoperative view of tumor and total aortic arch replacement. **a** Tumor invasion of AA. Arrowhead, LSA; arrow, LCCA. **b** Reconstruction of AA using branched woven shield vascular prosthesis. AA, aortic arch; BCA, brachiocephalic artery; LCCA, left common carotid artery; LSA, left subclavian artery

**Fig. 3:**
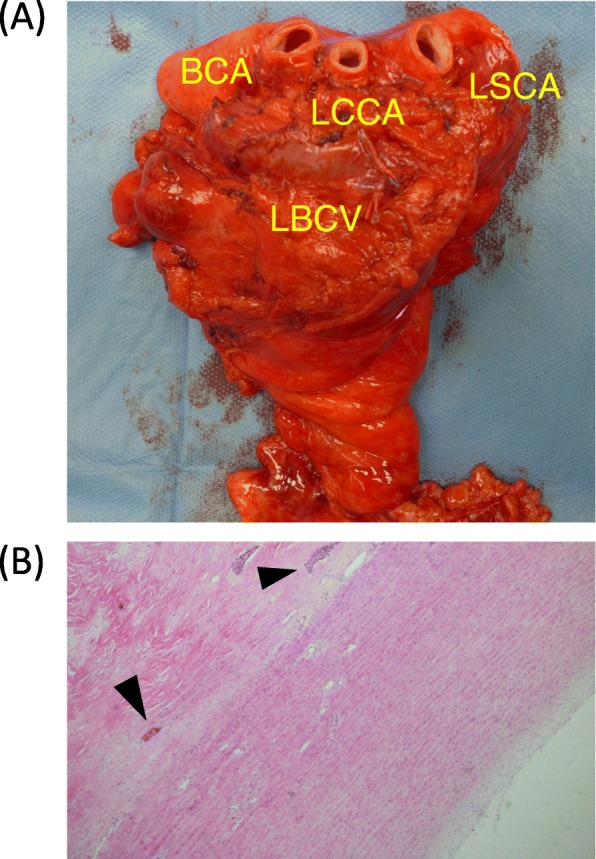
Macroscopic and microscopic findings of resected tumor. **a** Solid mass measuring 52 × 41 × 72 mm included LBCV, AA, LBCV, BCA, LCCA, and LSCA. **b** Hematoxylin and eosin staining (× 10) shows scattered viable cells (arrowhead) in aortic adventitia. BCA, brachiocephalic artery; LBCV, left brachiocephalic vein; LCCA, left common carotid artery; LSCA, left subclavian coronary artery

Chest X-rays revealed an abnormal shadow without symptoms such as hoarseness, in a 74-year-old man with a history of rheumatoid arthritis. Chest CT and MRI revealed an irregularly shaped mediastinal tumor measuring 60 × 60 × 55 mm that obstructed the left innominate vein and suspected invasion of the aortic arch. A CT-guided percutaneous needle biopsy revealed squamous cell carcinoma of the thymus that was considered unresectable. Since PET-CT and brain MRI ruled out metastatic lesions, such as mediastinal lymph node and brain metastases, the tumor was diagnosed as local advanced thymic carcinoma, Masaoka stage III, cT4N0M0 stage IIIB. The patient underwent six cycles of chemotherapy (three cycles each of ADOC and carboplatin/paclitaxel), which decreased the tumor to 55 × 55 × 50 mm on CT images (Fig. 1a, b) and the disease was defined as stable (SD), according to the RECIST criteria. Two months later, the patient was referred to us for consideration of surgical resection. We applied a radical surgical procedure through a median sternotomy and left lateral thoracotomy. Neither pleural/pericardial dissemination nor malignant effusion was evident. The tumor was located in the anterior mediastinum and had invaded the aortic arch around the root of the brachiocephalic artery (Fig. 2a), which rendered partial resection of the aortic arch unsuitable. Therefore, we replaced the entire aortic arch to achieve complete resection. The left brachiocephalic vein (LBCV), as well as the phrenic and vagal nerves, was resected; then, we encircled the distal sides of the brachiocephalic, left common carotid (LCCA), and left subclavian (LSCA) arteries, as well as the superior vena cava (SVC), ascending (AA), and descending aorta. Thereafter, a cardiopulmonary bypass (CPB) established with cannulation of the left femoral artery, right axillary artery, right femoral vein, and SVC was followed by deep hypothermic circulatory arrest with a bladder temperature of 23 °C. Thereafter, the tumor, aortic arch and three vessels were resected. The aortic arch was reconstructed using a 26-mm woven shield vascular prosthesis (Fig. 2b). The surgical duration was 678 min, blood loss was 3180 g, CPB duration was 175 min, including 81 and 31 min of cardiac and circulatory arrest, respectively. We did not use a cell salvage device. The postoperative course was uneventful, with durations of ventilation and stay in the ICU of 4 and 6 days, respectively. Figure 3a shows the macroscopic appearance of the resected tumor. The final pathological findings of the tumor were squamous cell carcinoma, ypT4N0M0, stage IIIB, and intra-tumoral fibrous stromal change surrounded by viable cancer cells. Viable cells were also scattered in the aortic adventitia (Fig. 3b), although the cut margin was pathologically negative. The patient remains alive and free of recurrence at over 3 years after surgery.

## Discussion

Thymic carcinoma is a relatively rare neoplasm that presents as late-stage disease, with reported 5-year survival rates of 30 to 60% [1]. Although complete surgical resection is an essential prognostic factor for overall survival [1, 3, 4], reported rates of complete resection vary from 20 to 88% because of aggressive invasion of the aorta or SVC [2]. We previously reported that multimodal treatment offered encouraging results and complete resection increases the survival rate of patients with advanced thymic carcinoma [5]. The National Cancer Care Network (NCCN) guidelines recommend that patients who have been carefully evaluated by board-certificated thoracic surgeons can undergo surgical resection, whereas those with unresectable and resectable tumors should be discussed and evaluated by a multidisciplinary team [6]. Optimal therapy remains undetermined due to the low prevalence of advanced thymic carcinoma. Complete resection is an independent prognostic indicator for patients with advanced thymic carcinoma. Therefore, although the surgical treatment is daunting, we consider that patients with thymic carcinoma who can medically tolerate surgical resection, who do not have apparent N2 disease, dissemination, or distant progression should be eligible for an attempt at surgical resection along with the infiltrated organs. Locally advanced thymic carcinoma involves vital structures in most patients with unresectable thymic carcinoma. The rationale for adding preoperative chemotherapy or chemoradiotherapy is an attempt to augment rates of complete resection and pathological responses. The resectability of remaining tumor after induction therapy is important for patients with locally advanced thymic carcinoma. Concurrent chemoradiotherapy is the standard strategy for locally advanced thymic cancer at our institution. However, the present patient had already been treated by six cycles of induction chemotherapy elsewhere 2 months before and had achieved SD status. His renal function was declining due to this therapy and he was unable to tolerate further chemotherapy. In addition, the patient might miss an opportunity for surgery if sequential radiation therapy failed. Therefore, we considered that additional chemotherapy or sequential radiotherapy was not suitable for this patient. We also considered that we could remove the tumor and replace the aortic arch without resecting other vital organs. Therefore, surgical resection included the tumor and infiltrated organs.

In conjunction with recent progress in the safety of cardiovascular surgical procedures and extracorporeal support techniques, we have resected both the AA and SVC under a CPB after chemoradiation therapy for locally advanced thymic carcinoma with successful outcomes [7]. Thus, we consider that locally advanced thymic carcinoma with involvement of the aortic arch can be included in the list of operative indications when complete resection is considered feasible through aortic arch replacement. Our patient did not develop any major complications such as cerebral infarction even though he underwent extended surgery after chemotherapy. Few studies have examined the outcomes of total aortic arch replacement in patients with advanced thymic carcinoma [8, 9] because infection involving vascular graft prostheses is associated with high morbidity. Thus, vascular reconstruction using vascular grafts with resection of the trachea or esophagus should be avoided.

## Conclusion

Even if the aortic arch has been invaded, an attempt at complete resection with aortic arch replacement might be a viable option for treating locally advanced thymic carcinoma.

## Data Availability

Not applicable

## Abbreviations

AA: Ascending aorta
ADOC: Cisplatin, doxorubicin, vincristine, cyclophosphamide
BCA: Brachiocephalic artery
CPB: Cardiopulmonary bypass
CT: Computed tomography
ICU: Intensive care unit
LBCV: Left brachiocephalic vein
LCCA: Left common carotid artery
LSCA: Left subclavian artery
MRI: Magnetic resonance imaging
NCCN: National Cancer Care Network
PET: Positron emission tomography
RECIST: Response evaluation criteria in solid tumors
SD: Stable disease
SVC: Superior vena cava
